# Characterization of Epstein-Barr virus (EBV)-infected cells in EBV-associated hemophagocytic lymphohistiocytosis in two patients with X-linked lymphoproliferative syndrome type 1 and type 2

**DOI:** 10.1186/2042-4280-3-1

**Published:** 2012-02-10

**Authors:** Xi Yang, Taizo Wada, Ken-Ichi Imadome, Naonori Nishida, Takeo Mukai, Mitsuhiro Fujiwara, Haruka Kawashima, Fumiyo Kato, Shigeyoshi Fujiwara, Akihiro Yachie, Xiaodong Zhao, Toshio Miyawaki, Hirokazu Kanegane

**Affiliations:** 1 Department of Pediatrics, Graduate School of Medicine and Pharmaceutical Science, University of Toyama, Toyama, Japan; 2 Division of Immunology, Children' s Hospital of Chongqing Medical University, Chongqing, China; 3 Department of Pediatrics, School of Medicine, Institute of Medical, Pharmaceutical and Health Sciences, Kanazawa University, Kanazawa, Japan; 4 Department of Infectious Diseases, National Research Institute for Child Health and Development, Tokyo, Japan; 5 Department of Pediatrics, Kurashiki Central Hospital, Kurashiki, Japan; 6 Department of Pediatrics, Tokyo Women's Medical University Medical Center East, Tokyo, Japan

**Keywords:** B cells, Epstein Barr virus, Hemophagocytic lymphohistiocytosis, X-linked lymphoproliferative syndrome

## Abstract

**Background:**

X-linked lymphoproliferative syndrome (XLP) is a rare inherited immunodeficiency by an extreme vulnerability to Epstein-Barr virus (EBV) infection, frequently resulting in hemophagocytic lymphohistiocytosis (HLH). XLP are now divided into type 1 (XLP-1) and type 2 (XLP-2), which are caused by mutations of *SH2D1A/SLAM-associated protein (SAP) *and *X-linked inhibitor of apoptosis protein (XIAP) *genes, respectively. The diagnosis of XLP in individuals with EBV-associated HLH (EBV-HLH) is generally difficult because they show basically similar symptoms to sporadic EBV-HLH. Although EBV-infected cells in sporadic EBV-HLH are known to be mainly in CD8^+ ^T cells, the cell-type of EBV-infected cells in EBV-HLH seen in XLP patients remains undetermined.

**Methods:**

EBV-infected cells in two patients (XLP-1 and XLP-2) presenting EBV-HLH were evaluated by in EBER-1 *in situ *hybridization or quantitative PCR methods.

**Results:**

Both XLP patients showed that the dominant population of EBV-infected cells was CD19^+ ^B cells, whereas EBV-infected CD8^+ ^T cells were very few.

**Conclusions:**

In XLP-related EBV-HLH, EBV-infected cells appear to be predominantly B cells. B cell directed therapy such as rituximab may be a valuable option in the treatment of EBV-HLH in XLP patients.

## Introduction

Hemophagocytic lymphohistiocytosis (HLH) is clinically characterized by prolonged fever, hepatosplenomegaly, hypertriglyceridemia, systemic hypercytokinemia and cytopenia [1]. HLH consists of primary (familial) and secondary (infection, lymphoma or autoimmune disease-associated) types. Approximately half of all infection-associated HLH cases involves the Epstein-Barr virus (EBV) [2]. Most cases of EBV-HLH are sporadic, but a few cases may present the first presentation of X-linked lymphoproliferative syndrome (XLP) [3]. XLP is a rare, inherited immunodeficiency that is characterized by an extreme vulnerability to EBV infection and shows variable clinical phenotypes, including severe or fatal EBV-HLH (60%), malignant B-cell lymphoma (30%), and progressive dysgammaglobulinemia (30%) [3]. The first genes that is responsible for XLP was identified as the *SH2D1A/SLAM-associated protein *(*SAP*) gene in 1998 [4-6], and mutations in the *X-linked inhibitor of apoptosis protein *(*XIAP*) gene can also lead to the clinical phenotype of XLP in 2006 [7]. XLP is now considered to comprise two distinct diseases, namely XLP-1 (SAP deficiency) and XLP-2 (XIAP deficiency).

In addition to B cells, EBV can infect other cell types, including epithelial cells, T cells and natural killer (NK) cells [8]. Studies have shown that activated T cells, particularly CD8^+ ^T cells, are the primary cellular target of EBV infection in sporadic EBV-HLH [9,10], which reflects the pathogenic role of EBV-infected CD8^+ ^T cells in sporadic EBV-HLH. Patients with sporadic EBV-HLH are usually treated with immunochemotherapy, including dexamethasone, cyclosporine A and etoposide, and this therapy can be curable [11]. In contrast, XLP-related EBV-HLH is usually refractory to immunochemotherapy [3]. It is possible that the poor response of XLP-related EBV-HLH to immunochemotherapy can be attributed to the type of EBV-infected cells in this disease, which may differ from the cell type that infected in sporadic EBV-HLH. We investigated the affected cell type in EBV infection of two XLP (XLP-1 and XLP-2) patients with EBV-HLH. Our results demonstrate that the predominant EBV target cells in XLP-related EBV-HLH are CD19^+ ^B cells, which appears to be distinct from sporadic EBV-HLH cases.

## Patients, materials and methods

### Patients

Three patients presented with clinical features of HLH, including persistent fever, hepatosplenomegaly, cytopenia, abnormal liver function, hyperferritinemia and elevated levels of soluble interleukin-2-receptor (Table 1). The clinical features of the patients fulfilled the diagnostic criteria for HLH [1], although hemophagocytosis in the bone marrow was not observed in patients 1 and 2. Patient 3 was previously reported as patient HLH3 [10]. The number of EBV-DNA copies in the peripheral blood was increased from the normal level of ≤ 1 × 10^2 ^copies/ml to 1.4 × 10^5^, 5.7 × 10^3 ^and 1.4 ×10^6 ^copies/ml in patients 1, 2 and 3, respectively. Blood samples from the patients were obtained using standard ethical procedures with the approval of the Ethics Committee of the University of Toyama, and an analysis of the *SH2D1A *and *XIAP *genes was performed. Patient 1 showed a one-nucleotide insertion (239_240insA) in the *SH2D1A *gene that resulted in a frameshift and a premature stop codon (80KfsX22). Patient 2 carried a two-nucleotides deletion (1021_1022delAA) in the *XIAP *gene that resulted in a frameshift and a premature stop codon (N341YfsX7). Patient 3 had no mutations in the *SH2D1A *or *XIAP *gene.

**Table 1:** Clinical and laboratory finding of the patients in this study

	Patient 1	Patient 2	Patient 3
Family history available	No	Yes	No

Age at the time of the study	4 years	21 months	16 months

Age at onset	3 years	17 months	16 month

Fever	Yes	Yes	Yes

Hepatomegaly	4 cm	5 cm	2.5 cm

Splenomegaly	2 cm	3 cm	1 cm

White blood cells (×10^9^/L)	11.6	6.36	3.03

Neutrophils (×10^9^/L)	1.61	3.915	0.56

Hemoglobin (g/dL)	8.1	9.6	7.5

Platelets (×10^9^/L)	95	56	30

LDH (IU/L)	449	1,693	1,698

AST (IU/L)	88	122	453

ALT (IU/L)	31	25	255

Ferritin (μg/L)	1,276	26,282	11,129

sIL-2R (U/mL)	3,162	2,880	14,334

IgG (mg/dL)	1,821	806	423

IgA (mg/dL)	302	124	32

IgM (mg/dL)	1,843	40	18

Whole blood EBV-DNA (copies/mL)	140,000	5,700	1,400,000

LDH, lactate dehydrogenase; AST, aspartate amino transferase; ALT, alanine amino transferase, sIL-2R, soluble interleukin-2 receptor; NA: not available.

### Cell preparation

Peripheral blood mononuclear cells (PBMCs) were isolated from the the patients using Ficoll-Hypaque gradient centrifugation. Lymphocytes were prepared from the PBMCs by depleting the monocytes using anti-CD14 monoclonal antibody (mAb)-coated magnetic beads (Becton Dickinson, San Diego, CA) [10]. The CD19^+ ^B cells, CD56^+ ^NK cells, CD4^+ ^T cells and CD8^+ ^T cells were purified by positive selection from the lymphocytes using the respective mAb-coated magnetic beads. The purity of each isolated cell population was assessed by flow cytometriy analysis, and each sorted population was found to be higher than 85% pure.

### *In situ *hybridization of EBVRNA

The presence of EBV was estimated by measuring the EBV-encoded small RNA 1 (EBER-1) mRNA using the *in situ *hybridization (ISH) method as described previously [10]. The sorted cells were cytocentrifuged onto silanized slides (Dako, Kyoto, Japan), and the presence of EBER-1 mRNA was determined by ISH using the alkaline phosphatase-conjugated EBER-1 antisense probe (5'-AGCAGAGTCTGGGAAGACAACCACAGACACCGTCCTCACC-3') or a sense probe.

### Quantitative PCR for EBV DNA

Quantitative PCR was performed using AmpliTaq Gold and a real-time PCR 7300 system (Applied Biosystems, Foster City, CA) as described previously [12]. The PCR primers for detecting EBV DNA were selected from within the *BALF5 *gene, which encodes the viral DNA polymerase. The primers for amplifying the *BALF5 *gene sequences were as follows: forward, CGGAAGCCCTCTGGACTTC, and reverse, CCCTGTT TATCCGATGGAATG. The TaqMan probe was FAM-TATACACGCACGAGAAATGCGCC-BFQ. The PCR conditions were as follows: denaturation at 95°C for 2 minutes, annealing at 58°C for 15 seconds, and extension at 72°C for 15 seconds, and the products were subjected to 45 cycles of PCR amplification. The EBV DNA copy number was considered to be significant when more than 500 copies/μg of DNA were observed.

### Flow cytometry analysis for the T cell receptor Vβ repertoire

Flow cytometry analysis of the T cell receptor (TCR) Vβ repertoire was performed as described previously [10]. In briefl, the PBMCs were incubated with the appropriate phycoerythrin-conjugated mAbs with specificity for TCR Vβ 1-23 (Immunotech, Marseille, France), fluorescein isothiocyanate-conjugated anti-CD8 (Becton Dickinson) and R-PE-Cy5-conjugated anti-CD4 (Dako) mAbs. The stained cells were analyzed using a flow cytometer. TCR Vβ expression is represented as the percentage of CD4^+ ^or CD8^+ ^cells for each receptor family.

## Results

**Figure 1:**
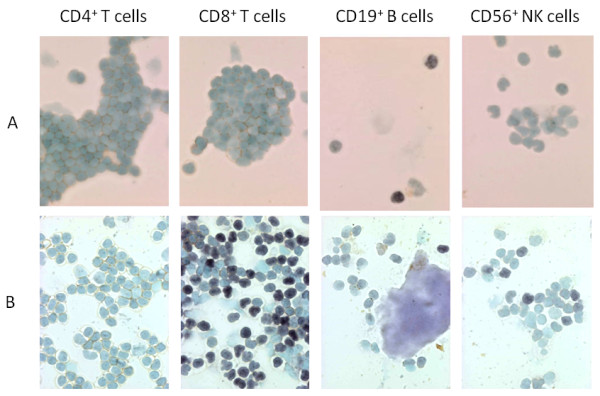
**Cytospin preparations showing EBER-1 *in situ *hybridization of the lymphocyte subpopulations**. Lymphocyte subpopulations from patients 1 and 3 were separated by magnetic bead sorting after immunostaining with anti-CD4, CD8, CD19 or CD56 mAbs. EBV infection in each subpopulation was determined using EBER-1 ISH. A, In patient 1, EBER-1-positive cells (shown by their dark nuclear staining) were detected in34.0% of the B cells but were not detected in CD4^+ ^T cells, CD8^+ ^T cells or CD56^+ ^NK cells (< 0.1% each). B, In patient 3, EBER-1-positive cells were observed in 75.5% of CD8^+ ^T cells, 2.8% of CD19^+ ^B cells, and 17.4% of CD56^+ ^NK cells, but not observed in CD4^+ ^T cells [10].

To determine the localization of EBV infection in the lymphocyte subpopulations of patient 1, CD4^+ ^T cells, CD8^+ ^T cells, CD19^+ ^B cells and CD56^+ ^NK cells were sorted using the immunomagnetic bead method and the presence of EBV was evaluated in each lymphocyte subpopulation by EBER-1 ISH (Figure 1A). EBER-1-positive cells were observed in 34.0% of the CD19^+ ^B cells, whereas the remaining lymphocyte subpopulations contained fewer than 0.1% EBER-1-positive cells. Therefore, the EBV-infected cells in patient 1 were almost exclusively CD19^+ ^B cells. In patient 3, EBER-1-positive cells constituted 75.5% of CD8^+ ^T cells, however, they were not detected among CD4^+ ^T cells and observed in a few of CD19^+ ^B cells and CD56^+ ^NK cells (2.8% and1 7.4%, respectively) (Figure 1B).

The viral loads in the CD4^+ ^T cells, CD8^+ ^T cells, CD19^+ ^B cells and CD56^+ ^NK cells in patient 2 were determined by quantitative PCR. The number of EBV DNA genome copies in the CD19^+ ^B cells was 1.8 × 10^4 ^copies/μg, and the copy number in the CD8^+ ^T cells was 1.0 × 10^3 ^copies/μg. The EBV DNA genome could not be detected in either the CD4^+ ^T cells or the CD56^+ ^NK cells that were isolated from patient 2.

**Figure 2:**
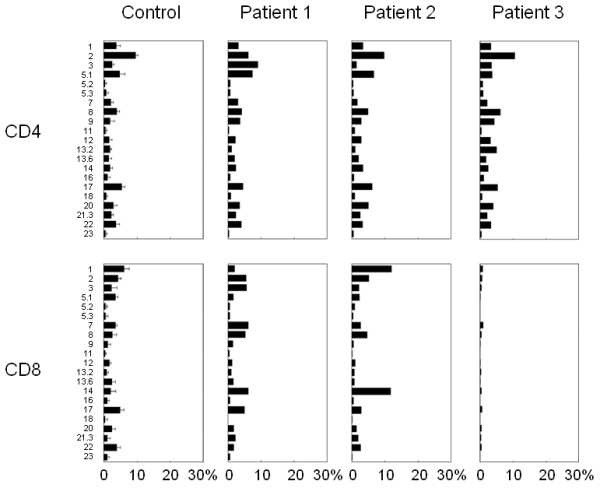
**The results of the flow cytometric analysis of TCR Vβ**. The expression profiles of the TCR Vβ subfamilies of patients 1, 2 and 3. The PBMCs were stained with mAbs for individual TCR Vβ, together with an anti-CD8 mAb. The percentage of the expression of each TCR Vβ within CD8^+ ^T cells was analyzed by flow cytometry.

Flow cytometry analysis of the TCR Vβ repertoire revealed a polyclonal pattern in patients 1 and 2 (Figure 2), which was in contrast to the skewed pattern that is most commonly seen in the CD8^+ ^T cells of patients with sporadic EBV-HLH [10]. No clonal dominance in CD8^+ ^T cells was demonstrated by mAb in patient 3, but TCR Vβ13.3 was predominantly found in the CD8^+ ^T cells by complentarity-determining region 3 spectratyping [10].

## Discussion

XLP is a severe and rare immunodeficiency disease that is characterized by an extreme vulnerability to EBV infection and frequently results in HLH [3]. XLP was first described as X-linked progressive combined immunodeficiency in 1975 by Purtilo et al. [13]. To better understand and reflect the pathophysiology of this disease, the term "X-linked lymphoproliferative disease or syndrome" has now been used. The first gene to be linked to XLP in 1998 was *SH2D1A *which is located on Xq25 and encodes the SAP [4-6]. Importantly, in 2006, a mutation in the gene that encodes the XIAP was identified as a second XLP-linked gene [7]. Thus, XLP can be divided into XLP-1 (SAP deficiency) and XLP-2 (XIAP deficiency). Most XLP patients present with EBV-HLH. Pachlopnik Schmid et al. [14] reported that the incidence of HLH in XLP-1 and XLP-2 is 55 and 76%, respectively. Currently, hematopoietic stem cell transplantation (HSCT) is the only curative therapy for XLP. Therefore, an early definitive diagnosis and immediate treatment are extremely important for both life-saving intervention and an improved prognosis for XLP patients.

EBV infects the majority of the adult population worldwide and persists in B cells throughout the lifetime of normal individuals, usually without causing disease. EBV is the most common trigger for both the XLP-1 and XLP-2 phenotypes. Prior to being exposed to EBV, most patients with XLP can tolerate infections by other agents, although *in vitro *studies have demonstrated defects of T cell-mediated and humoral immunity. During an acute EBV infection, XLP patients develop normal or high levels of anti-viral capsid antigen IgM antibodies but usually lack heterophile antibodies. Initially, these patients fail to develop EBV-specific cytotoxic T cells, and this results in a massive and overwhelming polyclonal B cell proliferation involving lymphoid and other tissues [8]. SAP binds 2B4, which is a surface molecule involved in activation of NK cell-mediated cytotoxity. Therefore, SAP-deficient patient shows that NK cell function is impaired, allowing B cell proliferation [15]. SAP has proapoptotic function, and contributes to the maintenance of T cell homeostasis and to the elimination of potentially dangerous DNA-damaged cells. Thus, the loss of this function could be responsible for the uncontrol T cell proliferation in acute EBV infection [16].

B cells are the usual cellular targets of EBV in a primary EBV infection such as infectious mononucleosis and in the sero-positive normal host [8]. After the interaction of the viral surface glycoproteins with the CD21 receptor, EBV entry into B cells is mediated by HLA class II and other co-receptors. However, in cases of sporadic EBV-HLH, EBV infects primarily T cells and NK cells [9,10,17]. The mechanism of T cell infection by EBV in HLH is still unclear, but one hypothesis is that, in specific situations, CD8^+ ^T cells express CD21, which can mediate EBV infection. Although T cells do not express the glycoprotein, they contain mRNA for CD21 [18]. In sporadic EBV-HLH cases, EBV infection into B cells is delayed but occurs during every case of cured EBV-HLH [17]. To the best of our knowledge, this is the first report of EBV infection status in two different types of XLP patients with EBV-HLH. The present study shows that the primary EBV-infected cells in XLP-related EBV-HLH are CD19^+ ^B cells and not T cells or NK cells, which are a primary target of EBV infection in sporadic EBV-HLH.

For decades, clinicians and investigators have been puzzled by the differential diagnosis between XLP and sporadic EBV-HLH when they encountered a young boy presenting with EBV- HLH. We believe the different EBV target cells can provide additional information to help discriminate between XLP and sporadic EBV-HLH. An evaluation of specific cell type that is infected by EBV should be considered when target therapy is applied. Most patients with sporadic EBV-HLH can achieve remission by immunochemotherapy; however, patients with XLP are usually refractory to this therapy. Recently, B cell-directed therapy using an anti-CD20 mAb (rituximab) was performed in patients with XLP-1 [19]. Two XLP patients who presented with acute EBV infection were successfully treated with rituximab and were free from EBV-HLH and lymphoma for a prolonged period. In addition, rituximab combined with methylprednisolone and intravenous immunoglobulin were administered to an XLP-1 patient with EBV-HLH, and the patient achieved a remission [20]. Patient 1 was also associated with EBV-associated encephalitis and lymphoproliferative disorder. The patient's lymphoproliferative disorder was treated with rituximab, but he died of the disease. Patient 2 was successfully treated with dexamethasone and immunoglobulin. Our data suggest that B cell target therapy can be a viable therapeutic option for an initial stage of EBV-HLH in both XLP-1and XLP-2 patients.

## Abbreviations

EBER: EBV-encoded small RNA; EBV: Epstein-Barr virus; HLH: Hemophagocytic lymphohistiocytosis; HSCT: Hematopoietic stem cell transplantation; ISH: *In situ *Hybridization; mAb: Monoclonal antibody; NK: Natural killer; PBMC: Peripheral blood mononuclear cells; SAP: SLAM-associated protein; TCR: T cell receptor; XIAP: X-linked inhibitor of apoptosis; XLP: X-linked lymphoproliferative syndrome.

## Competing interests

The authors declare that they have no competing interests.

## Authors' contributions

XY and HK wrote the manuscript. XY, TW, KI and NN performed the experimental studies. TM, MF, HK and FK managed the patients' care. SF, AY, XDZ and TM revised the manuscript. XY, TW and KI contributed equally to this study. All the authors read and approved the final manuscript.
